# The Influence of the Structural and Morphological Properties of WO_3_ Thin Films Obtained by PLD on the Photoelectrochemical Water-Splitting Reaction Efficiency

**DOI:** 10.3390/nano11010110

**Published:** 2021-01-06

**Authors:** Florin Andrei, Andreea Andrei, Ruxandra Birjega, Eduard Nicolae Sirjita, Alina Irina Radu, Maria Dinescu, Valentin Ion, Valentin-Adrian Maraloiu, Valentin Şerban Teodorescu, Nicu Doinel Scarisoreanu

**Affiliations:** 1 National Institute for Laser, Plasma and Radiation Physics, 077125 Magurele, Romania; florin.andrei@inflpr.ro (F.A.); andreea.chis@inflpr.ro (A.A.); ruxandra.birjega@inflpr.ro (R.B.); eduard.sirjita@inflpr.ro (E.N.S.); alina.calugar@inflpr.ro (A.I.R.); maria.dinescu@inflpr.ro (M.D.); valentin.ion@inflpr.ro (V.I.); 2 Faculty of Chemistry, University of Bucharest, 030018 Bucharest, Romania; 3 Faculty of Physics, University of Bucharest, 077125 Magurele, Romania; 4 National Institute for Material Physics, 077125 Magurele, Romania; maraloiu@infim.ro (V.-A.M.); teoval@infim.ro (V.Ş.T.)

**Keywords:** tungsten oxide, pulsed laser deposition, water-splitting, oxygen evolution reaction

## Abstract

Due to its physical and chemical properties, the n-type tungsten oxide (WO_3_) semiconductor is a suitable photoanode for water decomposition reaction. The responses of the photoelectrochemical PEC water-splitting properties as an effect of structural and optical changes of WO_3_ thin films, as well as the nature of electrolyte solutions, were studied in this work. The WO_3_ thins films have been obtained by pulsed laser deposition (PLD) on silicon (Si(001)) covered with platinum substrates using three different laser wavelengths. As the XRD (X-ray diffraction) and XTEM (cross-section transmission electron microscopy) analysis shows, the formation of highly crystalline monocline WO_3_ phase is formed for the film deposited at 1064 nm wavelength and poor crystalline phases with a large ordering anisotropy, characteristic of 2D structures for the films deposited at 355 nm and 193 nm wavelengths, respectively. The photogenerated current densities J_ph_ depend on the laser wavelength, in both alkaline and acidic electrolyte. The maximum values of the photocurrent density have been obtained for the sample prepared with laser emitting at 355 nm. This behavior can be correlated with the coherent crystallized atomic ordering that appear for long distances (10–15 nm) in the (001) plane of the monoclinic WO_3_ phase structure films obtained at 355 nm laser wavelength. All the samples show poor current density in dark conditions and they are very stable in both acidic and alkaline solutions. The highest photocurrent density value is obtained in acidic solution for the WO_3_ thin film prepared by 355 nm laser (29 mA/cm^2^ at 1.6 V vs. RHE (1.35 V vs. Ag/AgCl)).

## 1. Introduction

Presently, the global demand for energy critically is increasing, becoming one of humanity’s most important issues. Fossil fuels are among the most used sources for energy production. They are exhaustible resources and high energy demands will lead to complete consumption of these fuels. Furthermore, the use of this type of combustible is causing serious environmental problems such as the greenhouse effect and the pollution of the water and the air [1]. Hydrogen is considered one of the cleanest and the most suitable solution to solve the energy and environmental problems. This chemical element is among the most abundant in the universe and it can be used as fuel with no further emission of carbon oxides or other toxic gases [2]. However, the generated hydrogen needs costly advanced purification and nonetheless, the final product still contains large amounts of impurities [3]. In 1972, Fujishima et al. [4] have reported the first photoelectrochemical (PEC) water-splitting reaction with solar hydrogen production opening new opportunities in the photocatalytic field. The authors have used n-type TiO_2_ as photoanode and irradiation source emitting in the UV region.

Typically, two half reactions are involved in a photoelectrochemical cell, oxygen evolution reaction (OER) and hydrogen evolution reaction (HER) taking place at anode and cathode, respectively. Depending on the pH value of the electrolyte solution, the mechanism for HER can be dramatically changed. Therefore, in acidic medium H_2_ is produced mainly by protons reduction (2H_3_O^+^ + 2e^−^ → H_2_ +2H_2_O), while in alkaline environment hydroxide ions are first generated by the reduction of water (2H_2_O + 2e^−^ → H_2_ + 2OH^−^) [5]. Although the hydrogen is the desired reaction product, the OER is decisive for the overall efficiency of water-splitting reaction. The mechanism of OER is more complicated being a four electron-proton transfer process which can be the rate limiting step of the global redox reaction [6,7,8]. TiO_2_ is the most used photoelectrode for water-splitting reaction due to its low cost and enhanced resistance to photocorrosion. However, the overall efficiency of water-splitting reaction is narrow because of its high value of the band gap energy (~3.2 eV) and the rapid recombination of the photogenerated charges [9]. Many studies on both simple oxides such as ZnO [10], α-Fe_2_O_3_ [11], SnO_2_ [12], NiO [13] Cu_2_O [14] or multiple oxides as SrTiO_3_ [15], NaTaO_3_ [16], BiVO_4_ [17], LaFeO_3_ [18], CuFeO_2_ [19], CuBi_2_O_4_ [20], CuFe_2_O_4_ [21] designed and applied in photoelectrochemical water-splitting are reported in the literature. However, none of the listed materials can be used with satisfactory efficiencies for industrial scale requirements as photocatalyst for water-splitting reaction. Tungsten oxide (WO_3_) is an n-type semiconductor showing physical and chemical properties with applications in catalysis, gas sensors [22], window for photo-voltaic solar cells, electronic displays and color memory systems [23]. Also, it is suitable as photoanode for water decomposition reaction. However, it possesses a band gap value of ~2.6–2.7 eV offering the capability to absorb in both visible and ultra-violet region (λ ≤ 470 nm) [24]. Moreover, it is an excellent semiconductor for OER due to its valance band localization at positive potentials (2.8 V vs. NHE). For this reason, and due to poor kinetics at the surface of WO_3_, the photogenerated positive holes react with water generating hydrogen peroxide species (H_2_O_2_) besides O_2_ molecules. It was reported a decrease in the photogenerated current with the accumulation of hydrogen peroxide species [25,26]. Furthermore, WO_3_ has enhanced mobility of charge carriers and excellent chemical stability mainly in acid aqueous media (pH < 4) [27]. The surface morphology and the structure as well as the synthesis method of PEC photoelectrodes are critical parameters for global photoelectrochemical water-splitting efficiency. Different morphologies and efficiencies for bare WO_3_ have been reported by using numerous preparation methods: spin-coating (nanoparticles; IPCE ~66% at 320 nm) [28], solvothermal technique (nanowire; IPCE ~60% at 400 nm, nanoflake; IPCE ~60% at 400 nm) [29], hydrothermal method (nanorod; IPCE ~35% at 400 nm, plate-like films; IPCE ~66% at 360 nm, flake wall films; IPCE~ 40% at 400 nm, sandwich structured nanoplate; IPCE ~65% at 400 nm) [30,31], microwave-assisted hydrothermal method (nanoflowers; IPCE ~29% at 400 nm) [32] and pulsed laser deposition (PLD) (three similar to nanoporous; IPCE ~70% at 350–400 nm) [33,34]. High quality films can be manufactured by PLD which is an excellent deposition technique with great material transfer accuracy from target to substrate. The crystallinity and the stoichiometry of oxides are easily controlled by changing the atmosphere inside the deposition chamber [35,36]. Most of the reported studies on the PEC properties of WO_3_ thin films deposited by the PLD technique, but not limited to, have been focused on the monoclinic γ-WO_3_ phase, mostly because this polymorphic phase has a superior chemical stability and photoactivity [33,34,37]. However, the functional properties of the polymorphic γ-WO_3_ phase exhibit high sensitivity to structural defects (oxygen vacancies) or temperature. C. Fabrega et al. have reported high photocurrent density values, up to 3.1 mA/cm^2^ for the columnar shape γ-WO_3_/FTO films which favors the acidic 0.1 M H_2_SO_4_ electrolyte contact with the wall of the columns and contribute in this way to the overall charge transport for very thick films (up to 17 µm) [34]. The same approach is presented by S. Shin et al.: they obtained a tree-like nanoporous WO_3_ photoanode with enhanced OER, up to 9 times higher photocurrent density value as compared with dense WO_3_ films [33]. The preferred alignment of WO_3_ nanocrystals along the (010) crystallographic direction and the porous nature of the films made at different oxygen partial pressures being the main cause of the photocurrent density enhancements. The impact of the physical and chemical defects of WO_3_ thin films (Atomic Layer Deposition—ALD and sputtering produced) has also been presented by Y. Zhao et al., the inhibition of electrons and holes transport efficiencies due to both types of defects being the conclusion of the authors [38]. The thin films’ thickness problem in respect with the PEC efficiencies is also to consider, the charge carrier transport properties being highly limited by the thickness value of the semiconductor layer. The high recombination rates for Fe_2_O_3_ or BiVO_4_ materials at film’s thicknesses higher than 40–100 nm are common, limiting the light absorption potential of these materials [39,40,41].

To the mentioned structural, stoichiometric and transport properties issues, in the case of WO_3_ the pH value of the electrolyte is another topic to consider. The WO_3_ material has a high resistance to photocorrosion at pH < 4, in contract with Fe_2_O_3_, TiO_2_ or ZnO, and dissolves into aqueous alkaline solutions [42]. The use of acidic electrolyte is considered a risk from chemical safety point of view, hence researchers have tried to improve the stability of WO_3_ in neutral and alkaline environments (NaOH, KOH) [43]. Good results were obtained for WO_3_ nanostructures in electrolytes with pH ≥ 7 [33,44].

However, to our knowledge, there are no results on WO_3_ thin films obtained by PLD used as photoanode in alkaline electrolytes for photoelectrochemical water-splitting reaction. Moreover, no results are reported on the influence of the laser wavelength used in PLD method for photoelectrochemical properties in neither acidic, nor alkaline electrolyte solutions. Instead, there are many reports on the influence of the temperature and defects (oxygen vacancies) on the physicochemical properties of WO_3_ thin films [34]. M. Mai et al. reported WO_3_ thin films prepared at high oxygen partial pressure (0.13 mbar O_2_) that exhibit the best photoelectrochemical response, while the performances are decreasing with the reduction of the oxygen pressure. They have correlated the presented behavior with the crystallinity and the oxygen vacancies of the evaluated thin films [45].

In this work the influence of laser wavelengths used for the preparation by PLD of WO_3_ thin films on the PEC (OER) performance in acidic and alkaline electrolytes is reported. The use of different wavelengths for the preparation of WO_3_ thin films leads to different optical and structural properties affecting the overall efficiency of the photoelectrochemical water-splitting reaction. For a specific processing laser wavelength, nanostructured 2D columnar structure has been obtained and the overall photoelectrochemical behavior and the chemical stability in acidic as well alkaline diluted NaOH electrolyte solutions have been significantly enhanced.

## 2. Materials and Methods

### 2.1. Thin Films Deposition

The tungsten oxide (WO_3_) thin films were deposited by PLD on commercial silicon (Si(001)) covered with platinum (PtSi) substrates at different wavelengths and different laser fluences. The platinum is acting as bottom electrode for the electrical measurements. To perform the electrical contact with the platinum layer, a metallic mask was used to keep a partial undeposited area of the substrate. The substrates are placed in the PLD deposition chamber parallel with the rotating WO_3_ ceramic target. Before the irradiation, the pressure from the reactor chamber was reduced to ~10^−6^ mbar to remove the contaminants; after that, it was filled with a mixture of oxygen and argon (1:1) at 0.1 mbar. The producing of the thin films was performed in that gas mixture. During the deposition process, the substrates were heated to 600 °C with a heating step of 20 °C/min and after the deposition, the samples were cooled down to room temperature with 10 °C/min. The laser beam that hits the target was used at different wavelengths (193 nm from an ArF laser (Coherent, Göttingen, Germany), or 355 nm and 1064 nm generated by a Nd:YAG laser (Continuum, Pessac, France) with a repetition rate of 10 Hz. The laser fluences for the depositions of WO_3_ layers varied between 1.5 and 3 J/cm^2^, depending on the used wavelength. All the thin films were obtained as results of 20,000 laser pulses irradiating the target. For all experiments the distance between substrate and target was kept constant at 4 cm.

### 2.2. Characterization and Photoelectrochemical Measurements

The structural properties of the thin films were analyzed by X-ray diffraction (XRD) at room temperature with a PANalytical X’Pert MPD diffractometer (Almelo, The Netherlands) using the CuKα radiation (0.15418 nm) and a large acquisition time of 40 s/0.02 deg. The PANalytical X’Pert MPD diffractometer is θ-θ system and is equipped with a horizontal fixed sample stage. Optical characterization was performed using a Woollam V-VASE ellipsometer (J.A. Woollam CO., INC., Lincoln, NE, USA), equipped with a monochromator HS-190, for a spectral range between 250 nm and 1700 nm, with a measuring step of 2 nm, with an incident angle of light of 70 degrees. The Cross-sectional Transmission Electron Microscopy (XTEM) measurements were performed using a JEOL ARM200F electron microscope (Joel, Tokyo, Japan). The cross-section specimens were prepared using the classical tripod mechanical thinning and polishing method and finally by ion thinning in a Gatan device. The photoelectrochemical measurements were performed in a three-electrode system configuration quartz cell connected to an Autolab potentiostat/galvanostat. The counter electrode was a helicoidal Pt wire, Ag/AgCl (3.5 M KCl) used as reference electrode and the working electrode was the obtained thin film itself. Two different aqueous solutions with different pH values, NaOH (pH = 10; 10^−4^ M) and H_2_SO_4_ (pH = 0.6; 0.5 M), were used as electrolytes. In the case of the working electrodes, the electrical connection was done with the Pt layer from the substrate, using a conductive wire and silver paste. To avoid the short-circuit, all the samples were electrically insulated using an epoxy resin and only the surface of the thin films was directly contacted with the basic electrolyte solution. Linear sweep voltammetry’s (LSV) with a small scanning rate of 5 mV/s, to avoid the effect of concentration polarization, were first recorded in dark and under chopped irradiation conditions using a 404 nm laser diode (~5 mW output power). The power of the laser beam at the sample surface was ~4.4 mW, mentioning that the absorption of the electrolyte solution was considered to be negligible. The stability of the photoelectrodes (thin films) in both alkaline and acidic media was studied using chronoamperometry, an electrochemical technique which offers the possibility to measure the photogenerated current in time at a fixed potential value.

The structural properties of the thin films were analyzed by X-ray diffraction (XRD) at room temperature with a PANalytical X’Pert MPD diffractometer (Almelo, The Netherlands) using the CuKα radiation (0.15418 nm) and a large acquisition time of 40 s/0.02 deg. The PANalytical X’Pert MPD diffractometer is θ-θ system and is equipped with a horizontal fixed sample stage. Optical characterization was performed using a Woollam V-VASE ellipsometer (J.A. Woollam CO., INC., Lincoln, NE, USA), equipped with a monochromator HS-190, for a spectral range between 250 nm and 1700 nm, with a measuring step of 2 nm, with an incident angle of light of 70 degrees. The Cross-sectional Transmission Electron Microscopy (XTEM) measurements were performed using a JEOL ARM200F electron microscope (Joel, Tokyo, Japan). The cross-section specimens were prepared using the classical tripod mechanical thinning and polishing method and finally by ion thinning in a Gatan device. The photoelectrochemical measurements were performed in a three-electrode system configuration quartz cell connected to an Autolab potentiostat/galvanostat. The counter electrode was a helicoidal Pt wire, Ag/AgCl (3.5 M KCl) used as reference electrode and the working electrode was the obtained thin film itself. Two different aqueous solutions with different pH values, NaOH (pH = 10; 10^−4^ M) and H_2_SO_4_ (pH = 0.6; 0.5 M), were used as electrolytes. In the case of the working electrodes, the electrical connection was done with the Pt layer from the substrate, using a conductive wire and silver paste. To avoid the short-circuit, all the samples were electrically insulated using an epoxy resin and only the surface of the thin films was directly contacted with the basic electrolyte solution. Linear sweep voltammetry’s (LSV) with a small scanning rate of 5 mV/s, to avoid the effect of concentration polarization, were first recorded in dark and under chopped irradiation conditions using a 404 nm laser diode (~5 mW output power). The power of the laser beam at the sample surface was ~4.4 mW, mentioning that the absorption of the electrolyte solution was considered to be negligible. The stability of the photoelectrodes (thin films) in both alkaline and acidic media was studied using chronoamperometry, an electrochemical technique which offers the possibility to measure the photogenerated current in time at a fixed potential value.

## 3. Results and Discussion

### 3.1. Structural Characterization

#### 3.1.1. XRD Measurements

**Figure 1:**
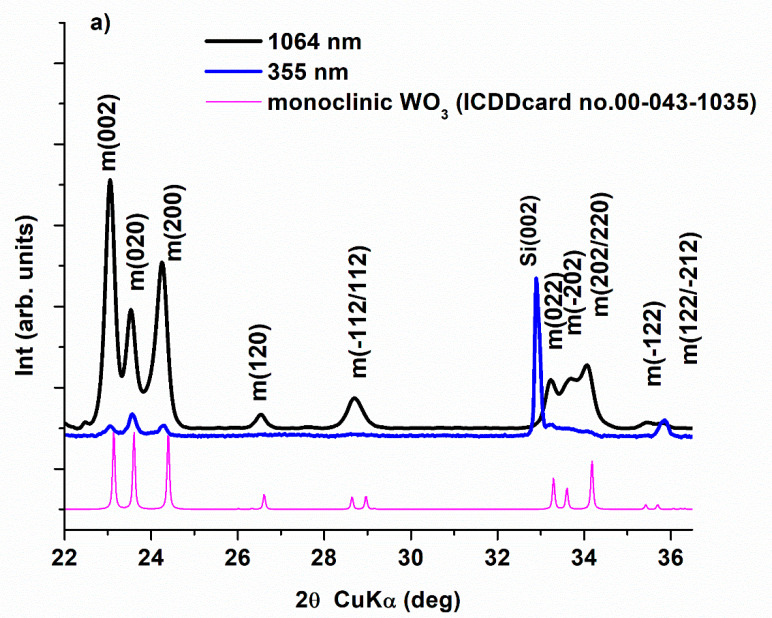

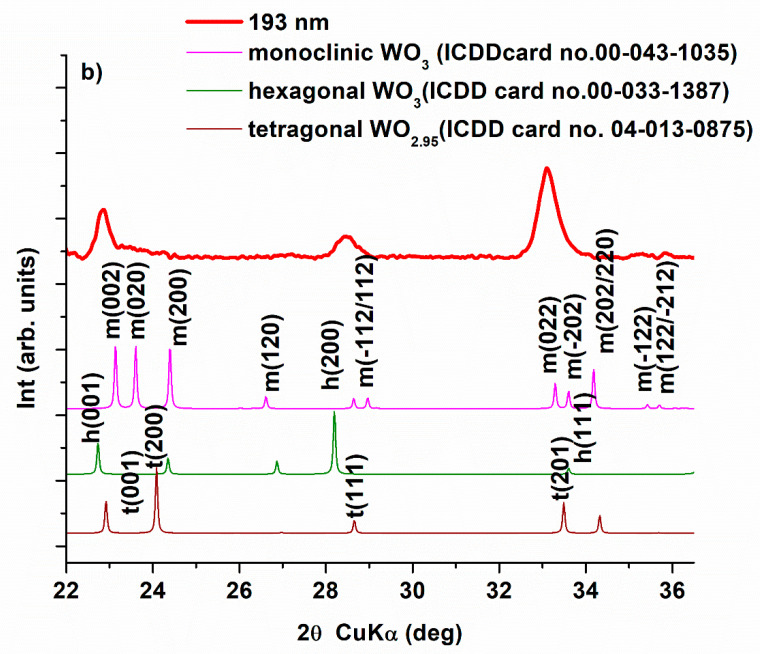
(**a**) XRD diffractograms of WO_3_ thin films deposited onto PtSi substrate at different laser wavelengths: 1064 nm (black line) and 355 nm (blue line) and, (**b**) XRD diffractogram of the WO_3_ film deposited at 193 nm (red line).

**Figure 2:**
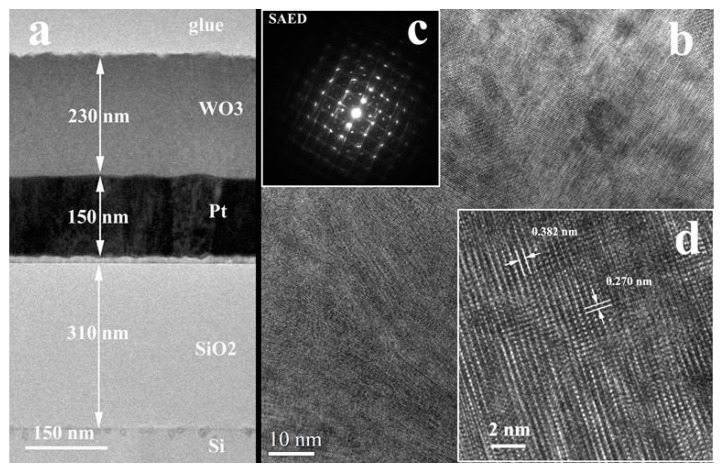
XTEM images of the WO_3_ film deposited on Pt/SiO_2_/Si substrate using the 193 nm laser radiation: (**a**)—Low magnification TEM image of the sample cross-section; (**b**)—poor-crystallized columnar grain structure; image collected in the middle of the film thickness; (**c**)—SAED of the columnar grain structure; (**d**)—HRTEM detail of the WO_3_ columnar grain structure.

The XRD patterns of the WO_3_-thin films (Figure 1) show the formation of a highly crystalline monoclinic (γ) WO_3_ phase for the film deposited at 1064 nm and of poor crystalline monoclinic phase for the film deposited at 355 nm. The mean crystallites sizes/coherence lengths determined via the Scherrer formula are 35 nm for the film at 1064 nm and only about 20 nm for the film at 355 nm. The kinematically forbidden Si(002) reflection appearing through double diffraction effects is visible in the film at 355 nm due to the better orientation of its substrate regarding the Si(004) reflection in the x-ray beam. The XRD patterns of the film deposited at 193 nm revealed only three broad peaks and a halo peak around 2*θ* = 24°. The two main peaks at 2*θ* = 22.86° and 2*θ* = 33.13° correspond to the interplanar distances of 3.89 Å and 2.70 Å, respectively, which are disclosed as well by the HTREM image of this film (see Figure 2d). The XTEM analysis also reveals a preferential *c*-axis oriented growth with an important ordering anisotropy along this axis. Therefore, some of reflections will be missing or will be ill-defined making the precisely assignment of the crystalline phases quite difficult. A search of the ICDD’s reference card files of the various crystalline structures shows possible matches with hexagonal WO_3_ (ICDD card no.00-033-1387) or tetragonal WO_2.95_ (ICCD card no.04-013-0875) with a preferred *c*-oriented growth. A monoclinic structure with a highly *c*-axis oriented growth could be also considered. This interpretation is supported by previous reports. Y. S. Zhou et al. [46] show that the deposition for a series of WO_3_ films via PLD at 193 nm on Si(001) substrates at different temperatures the XRD patterns evolves from an amorphous structure at 200 °C, through a pattern with a sharp peak at 2*θ* = 23.1° accompanied by two small peaks at 2*θ* = 28.8° and 33.3° at 300 °C, to a pattern with more defined peaks as at 2*θ* = 23.6°, characteristic of a monoclinic structure at 500 °C and 600 °C. A. Palla-Papavlu et.al [47] reported also on a preferential (002) direction growth of a monoclinic-WO_3_ film obtained by PLD deposition on Pt electrodes at 600 °C at 193 nm. 

#### 3.1.2. XTEM Measurements

**Figure 3:**
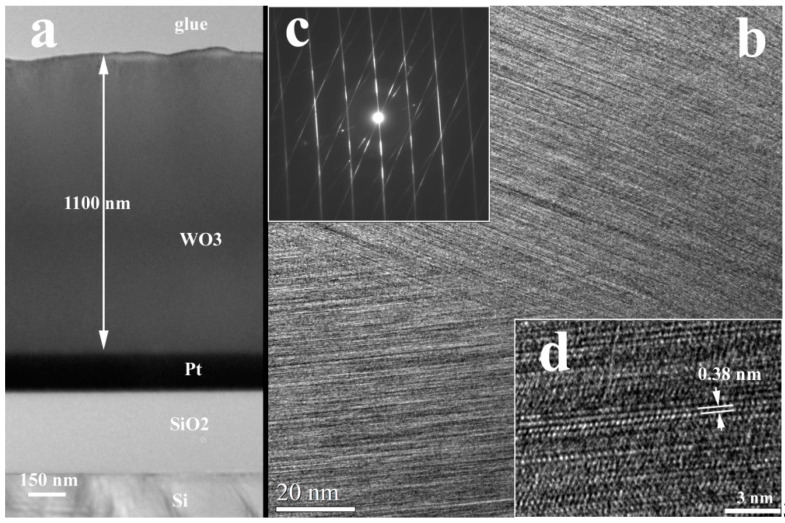
XTEM images of the WO_3_ film deposited on Pt/SiO_2_/Si substrate using the 355 nm laser radiation; (**a**)—Low magnification TEM image of the sample cross-section; (**b**)—columnar grain structure of the film; (**c**)—SAED of the columnar grain structure; (**d**)—HRTEM detail of the WO_3_ columnar grain structure, showing the 2D crystalline ordering.

**Figure 4:**
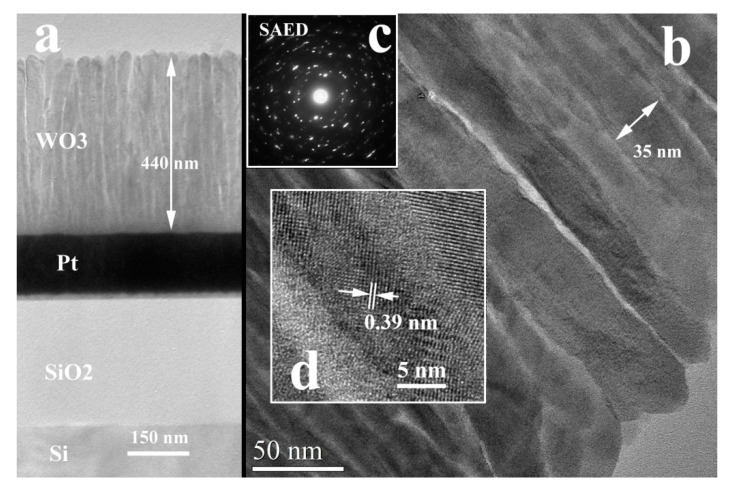
XTEM images of the WO_3_ film deposited on Pt/SiO_2_/Si substrate using the 1064 nm laser radiation; (**a**)—Low magnification TEM image of the sample cross-section; (**b**)—crystallized columnar grain structure of the film; (**c**)—SAED of the columnar grain structure; (**d**)—HRTEM detail of the WO_3_ columnar grain structure.

Figure 2, Figure 3 and Figure 4 present the low magnification XTEM images of the samples obtained at different laser radiation wavelengths: 193 nm, 355 nm, and 1064 nm. The structure of the WO_3_ films deposited with the 193 nm and 355 nm laser radiations shows a crystallization with a large ordering anisotropy, similar to a 2D crystalline structure. This means that the coherent crystallized atomic ordering appears for long distances (10–15 nm) in the (001) plane of the monoclinic WO_3_ phase structure. The crystalline ordering perpendicular to this plane is very poor, extending only about 1 nm, meaning 2 or 3 planar distances. In the Selected Area Electron Diffraction (SAED) pattern of this structure appear long sticks in the place of reflections spots, especially for the films deposited with 355 radiation. The interplanar distances of about 0.38 nm (between 0.38 and 0.39 nm) are present in all HRTEM images of the columnar grain WO_3_ structures. This distance appears in all known structures of the WO_3_: 0.388 nm for (001) WO_3_ tetragonal (ICDD card no 01-089-1287; 0.382 nm for (002)) WO_3_ monoclinic (ICCD no. 01-087-2387); 0.383 nm for (002) WO_3_ tetragonal; etc. The packing of this atomic plane with a lattice distance of 0.38 nm is distorted in out structures and this can be associated also with the nonstoichiometry of the films. Some of the tungsten oxide, for example W_18_O_49_ and others, contain the same lattice planes, but with different monoclinic angles. This means that if we have some loss of oxygen, this can produce a variation of the packing of this atomic layer and a loss of the crystalline coherence perpendicular to this plane. However, this atomic plane remains in parallel position in all the columnar grain.

The difference between the films deposited with the 193 nm and 355 nm radiations is very consistent. First is the final thickness of the films, which is 230 nm (for the 193 nm) and 1100 nm (for the 355 nm). In the second case, some parts of the films show a thickness of about 500–600 nm, showing a local deposition rate variation. This feature can be possible explained by the different rate of grow of the different crystallographic orientation of the columnar grains at the film surface. It is also clear that the crystallographic coherence is better in the case of the films deposited with the 193 nm radiation compared to the films deposited with the 355 nm radiation.

The films deposited with the 1064 nm radiation show a WO_3_ structure for the columnar grain as is shown in Figure 4b. The indexing of some SAED from the different column grains shows the presence of the monoclinic and tetragonal structures (see Figure 4c). It is interesting to mention that the energy-dispersive X-ray spectroscopy (EDX) measurements indicate a near WO_3_ stoichiometry for all the samples.

### 3.2. Optical Characterization

Optical investigations were realized by ellipsometry using a J.A. Wollam Spectroscopic Ellipsometer (J.A. Woollam CO., INC., Lincoln, NE, USA). This technique is based on characterizations of the complex refractive index of materials [48]. The extraction of the extinction coefficients and absorption coefficients at 404 nm irradiation wavelength is presented in Table 1. The absorption coefficient obtained for the sample prepared with the ArF excimer laser (emitting at 193 nm) is similar to that obtained for WO_3_ thin films prepared by KrF excimer laser. However, the values of the absorption coefficients for the WO_3_ thin films prepared by Nd:YAG laser are slightly higher [34].

### 3.3. Photoelectrochemical Measurements

WO_3_ thin films were used as photoanodes in photoelectrochemical water-splitting to evaluate OER photocurrent values as a function of the laser wavelength as well as the nature of the electrolyte solutions. First, the electrochemical response of thin films has been studied in the absence of illumination in the 0.1–1.4 V vs. Ag/AgCl potential range—see Figure S1 Supporting Information. In both alkaline and acidic electrolytes, all the samples show small values of current densities—not showed here. In the case of WO_3_/PtSi sample prepared with Nd:YAG laser emitting at 1064 nm, there is a slight increase of the current density, but the values are still very low. There is clear that none of the measured samples present catalytic activity in dark conditions. Moreover, no significant generated current that could be correlated with the leakage current or to other redox processes which could take place in the electrolyte solutions appears.

**Figure 5:**
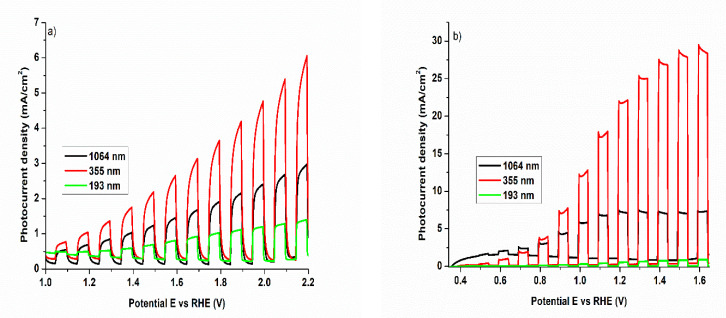
Potentiodynamic measurements under chopped irradiation on WO_3_/PtSi thin films manufactured at different laser wavelengths in (**a**) alkaline electrolyte: NaOH and (**b**) acidic electrolyte: H_2_SO_4._

The chopped potentiodynamic measurements indicate a n-type semiconductor behavior of WO_3_ samples, in that the anodic photocurrent increases with the positive potential, as presented in Figure 5. The potential was converted as a function of RHE using the following formula: E_(RHE)_ = E_Ag/AgCl_ + 0.059 pH + E^o^_Ag/AgCl_, where E_Ag/AgCl_ is the working potential; pH—is the pH value of the electrolyte; E^o^_Ag/AgCl_ = + 0.205 V (we have used an Ag/AgCl 3.5 KCl reference electrode). A clear tendency is observed in photogenerated current densities J_ph_ as a function of laser wavelength, in both alkaline (Figure 5a) and acidic (Figure 5b) media. This tendency is independent on the electrolyte nature, the maximum values of the photocurrent density being obtained for the sample prepared with laser emitting at 355 nm. This behavior can be correlated with the coherent crystallized atomic ordering appear for long distances (10–15 nm) in the (001) plane of the monoclinic WO_3_ phase structure. It worth mentioning here—as stated above—that the crystalline ordering perpendicular to this plane is very low, extended only about 1 nm. It is obvious that this structural (001) 2D nanodomain ordering is improving the transfer and the separation rate of the photogenerated species, leading to a higher photoelectrochemical efficiency [49].

In acidic environment, all the samples show higher photocurrent values compared to alkaline solution, with a maximum value of ca. 29 mA/cm^2^ at 1.6 V vs. RHE (1.35 V vs. Ag/AgCl) for the sample prepared with laser irradiating at 355 nm. These results are contradictory to other studies on different oxides (TiO_2_ [50], ZnO [51], BiVO_4_ [52]), where the photoelectrochemical performance increases with the pH value. Despite high instability of WO_3_ in alkaline media [53], our WO_3_ thin films show an excellent photoelectrochemical response, with no noticeable leakage currents or short circuits in the measured potential range. However, the maximum value of the photocurrent density obtained in alkaline electrolyte is ca. 6 mA/cm^2^, which is much smaller compared to the results obtained in acidic condition. To check if the electrolyte conductivity is the main factor which decreases the photocurrent density, the samples were tested in a 0.5 M Na_2_SO_4_ solution adjusted at pH = 10. It was observed that the magnitude of the photocurrent in both alkaline solutions remains constant. However, by increasing the concentration of alkaline electrolyte, the transient photocurrent increases as well—see Figure S2 Supporting Information. The lowering of the photocurrent in the alkaline medium can be attributed to the slow charge transfer to the surface, as well as the fast free charges recombination [43]. Moreover, in strongly NaOH media, peroxide species, and sodium compounds are predisposed to be formed, which possibly interfered with light absorbance, leading to a decrease of the photocurrent [27,54]. The calculated Absorbed Photon to Current Efficiencies (*APCE*) at different applied potentials for WO_3_/PtSi thin film in both acidic and alkaline media are resumed in Table S1 (see Supporting Information). We consider that *APCE* is less subjected to input parameters errors and it can be calculated by using the formula:

$$APCE=\;\frac{J\left(\mathrm{mA}\cdot {\mathrm{cm}}^{-2}\right)\times 1239.8\;\left(V\cdot \mathrm{nm}\right)}{P_{\lambda }\left(\mathrm{mW}\cdot {\mathrm{cm}}^{-2}\right)\;\times \;\lambda \left(\mathrm{nm}\right)\;\times A}$$

with the absorptance calculated as shown below from the extinction coefficients (k) measured by spectroscopic ellipsometry and the film thickness (*t*) calculated by XTEM: $A=\;1-e^{-\frac{4\pi k\cdot t}{\lambda }}$.

No other photoelectrochemical results on PLD prepared samples using our reported wavelengths are published to date. Nonetheless, the absolute efficiencies for water-splitting reaction of these WO_3_ samples are to be tested using, in the near future, a gas chromatograph system in conjunction with PEC system.

Considering the influence of the films thickness over the functional photoelectrochemical properties of WO_3_ films, the photocurrent density response of 355 nm laser produced WO_3_ films as a function of thickness is not yet clear. We have deposited and characterized a 355 nm laser wavelength WO_3_/Pt/Si film, with a thickness in between the 193 nm and 1064 nm laser wavelengths produced ones, just to demonstrate that this PEC performance versus film thickness is the case of 355 nm laser produced WO_3_ films, is far for being clarified—please see Supporting Information Figure S3. For a thickness of around 330 nm, the WO_3_ sample exhibiting similar or even better photocurrent density values on the same range of potential E vs. Ag/AgCl. The sample is thick enough to ruled-out any structural strain induced by the substrate lattice, and thin enough as respect to the 1064 nm film (440 nm). If one considers that the absorption coefficient for WO_3_ material is low compared with similar materials, then thicker films are needed to fully absorption of the solar light. However, this is not the case for the 355 nm laser produced WO_3_ film. These aspects, involving the effect of the film’s thickness over the photoelectrochemical performances are to be clarified, as the 193 and 355 nm laser produced films are characterized by a highly anisotropic coherent crystallinity.

**Figure 6:**
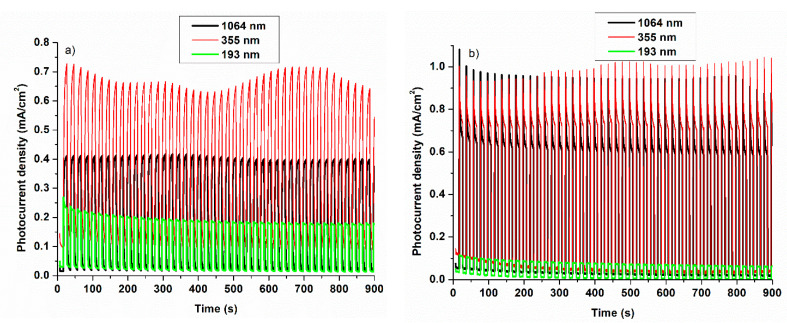
Potentiostatic measurements of WO_3_/PtSi thin films manufactured at different laser wavelengths in (**a**) alkaline electrolyte: NaOH and (**b**) acidic electrolyte: H_2_SO_4_ (0.4 V vs. Ag/AgCl).

Figure 6 presents the potentiostatic measurements in alkaline (Figure 6a) and acidic (Figure 6b) solutions. The measurements are performed at 0.4 V vs. Ag/AgCl. All the samples show excellent stabilities in both electrolytic solutions. No major differences are observed between the samples prepared at 1064 nm and 355 nm in the acidic electrolyte. Also, as can be observed the photocurrent obtained for the sample prepared at 193 nm is higher in alkaline electrolyte, with a maximum value of ca. 0.25 mA/cm^2^.

## 4. Conclusions

In this study, it is demonstrated that the overall photoelectrochemical water-splitting efficiency is strongly dependent on the structural properties of WO_3_/PtSi thin films, prepared by PLD using different laser wavelengths. As expected, the generated J_ph_ is vulnerable to the chemical nature of the electrolytes. All the thin films show no significant current densities in the absence of the irradiation in both alkaline and acidic media. A clear tendency is observed for the photogenerated current densities J_ph_ of the samples obtained as a function of laser wavelength, in both alkaline and acidic media. The thin films prepared at 355 nm with a poor crystalline monoclinic phase but with 2D columnar ordering, is showing the best J_ph_, with a maximum value of ca. 29 mA/cm^2^ at 1.6 V vs. RHE (1.35 V vs. Ag/AgCl) in acidic media. This behavior can be correlated with the coherent crystallized atomic ordering appearing for long distances (10–15 nm) in the (001) plane of the monoclinic WO_3_ phase structure, combined with very low crystalline ordering perpendicular to this plane. The photocurrent density decreases in the alkaline electrolyte because of the slow charge transfer of charge carriers to the electrode-electrolyte interface and their fast recombination. All the samples show excellent stabilities in both acidic and alkaline solutions, the stability being still preserved after 900 s. Moreover, the versatility of PLD for growing nanostructured tungsten oxide thin films with different structural properties was proved.

## Figures and Tables

**Table 1:** The values of extinction coefficient and the absorption coefficient for a specific value of spectroellipsometry (SE) measured wavelength (404 nm).

Laser Wavelength (nm)	K_404 nm_ (dimensionless)	Absorption Coefficient α_404 nm_ × 10^4^ (cm^−1^)
193	0.06142	1.91
355	0.11657	3.62
1064	0.10747	3.34

## Data Availability

Data is contained within the article or supplementary material.

## Supplementary Materials

The following are available online at https://www.mdpi.com/2079-4991/11/1/110/s1, Figure S1: Linear sweep voltammetry without irradiation on WO_3_/PtSi thin films manufactured at different laser wavelengths in (a) alkaline electrolyte: NaOH and (b) acidic electrolyte: H_2_SO_4_, Figure S2: Potentiondynamic measurements under chopped irradiation on WO_3_/PtSi thin films manufactured at 1064 nm laser wavelengths in different concentration of alkaline electrolytes, Figure S3: Potentiondynamic measurements under chopped irradiation on WO_3_/PtSi thin films manufactured at 355 nm laser wavelengths with different thickeness, Table S1: APCE at 404 nm for WO_3_/PtSi thin films.

## References

[1] Yogesh M., Seyed E.H., Brayden B., Hisham A., Bhi T.F.S., Turaj A., John K. (2019). Hydrogen fuel cell vehicles; Current status and future prospect. Appl. Sci..

[2] Takashi H., Kazunari D. (2019). Reaction systems for solar hydrogen production via water splitting with particulate semiconductor photocatalysts. Nat. Catal..

[3] Konieczny A., Mondal K., Wiltowski T., Dydo P. (2008). Catalyst development for thermocatalytic decomposition of methane to hydrogen. Int. J. Hydrog. Energy.

[4] Fujishima A., Honda K. (1972). Electrochemical photolysis of water at a semiconductor electrode. Nature.

[5] Michael G.W., Emily L.W., James R.M., Shannon W.B., Qixi M., Elizabeth A.S., Nathan S.L. (2010). Solar water splitting cells. Chem. Rev..

[6] Ju S.K., Byunghoon K., Hyunah K., Kisuk K. (2018). Recent progress on multimetal oxide catalysts for the oxygen evolution reaction. Adv. Energy Mater..

[7] Congling H., Lei Z., Jinlong G. (2019). Recent progress made in the mechanism comprehension and design of electrocatalysts for alkaline water splitting. Energy Environ. Sci..

[8] Wang W., Xu X., Zhou W., Shao Z. (2017). Recent progress in metal-organic frameworks for applications in electrocatalytic and photocatalytic water splitting. Adv. Sci..

[9] Wang G., Wang H., Ling Y., Tang Y., Yang X., Fitzmorris R.C., Wang C., Zhang J., Li Y. (2011). Hydrogen-treated TiO_2_ nanowire arrays for photoelectrochemical water splitting. Nano Lett..

[10] Wang B.S., Li R.Y., Zhang Z.Y., Wang X., Wu X.L., Cheng G.A., Zheng R.T. (2019). An overlapping ZnO nanowire photoanode for photoelectrochemical water splitting. Catal. Today.

[11] Xiaobo C., Haifeng Z., Qin P., Jialin B., Zuofeng C., Chuanwei C. (2019). 3D ordered urchin-like TiO_2_@Fe_2_O_3_ arrays photoanode for efficient photoelectrochemical water splitting. Appl. Surf. Sci..

[12] Zemin Z., Caitian G., Zimao W., Weihua H., Yaling W., Wenbing F., Xiaodong L., Erqing X. (2016). Toward efficient photoelectrochemical water-splitting by using screw-like SnO_2_ nanostructures as photoanode after being decorated with CdS quantum dots. Nano Energy.

[13] Chenyan H., Kenneth C., Yihua Z., Wey Y.T. (2014). Efficient photoelectrochemical water splitting over anodized p-type NiO porous films. ACS Appl. Mater. Interfaces.

[14] Luo J., Steier L., Son M.K., Schreier M., Mayer M.T., Grätzel M. (2016). Cu_2_O nanowire photocathodes for efficient and durable solar water splitting. Nano Lett..

[15] Yang L., Lei X., Yan L., Rong Y., Jianglan Q., Yaoqi L., Xingguo L. (2008). Synthesis and high photocatalytic hydrogen production of SrTiO_3_ nanoparticles from water splitting under UV irradiation. J. Power Sources.

[16] Akihiko K. (2006). Development of photocatalyst materials for water splitting. Development of Photocatalyst Materials for Water Splitting. Int. J. Hydrog. Energy.

[17] Liu Q., Mo R., Li X., Yang S., Zhong J., Li X. (2019). Cobalt phosphate modified 3D TiO_2_/BiVO_4_ composite inverse opals photoanode for enhanced photoelectrochemical water splitting. Appl. Surf. Sci..

[18] Andrei F., Boerasu I., Moldovan A., Dinescu M., Ion V., Mihailescu C., Scarisoreanu N.D., Leca V. (2019). The effects of the oxygen content on the photoelectrochemical properties of LaFeO_3_ perovskite thin films obtained by pulsed laser deposition. Appl. Phys. A.

[19] Carrie G.R., Yiseul P., Kyoung S.C. (2012). Electrochemical synthesis of p-type CuFeO_2_ electrodes for use in a photoelectrochemical cell. J. Phys. Chem. Lett..

[20] Berglund S.P., Abdi F.F., Bogdanoff P., Chemseddine A., Friedrich D., van de Krol R. (2016). Comprehensive evaluation of CuBi_2_O_4_ as a photocathode material for photoelectrochemical water splitting. Chem. Mater..

[21] Park S., Baek J.H., Zhang L., Lee J.M., Stone K.H., Cho I.S., Guo J., Jung H.S., Zheng X. (2019). Rapid flame-annealed CuFe_2_O_4_ as efficient photocathode for photoelectrochemical hydrogen production. ACS Sustain. Chem. Eng..

[22] Pokhrel S., Simion C.E., Teodorescu V.S., Barsan N., Weimar U. (2009). Synthesis, mechanism, and gas-sensing application of surfactant tailored tungsten oxide nanostructures. Adv. Funct. Mater..

[23] Filipescu M., Orlando S., Russo V., Lamperti A., Purice A., Moldovan A., Dinescu M. (2007). Pulsed laser deposition of nanostructured MoS_3_/np-Mo//WO_3_−y Hybrid catalyst for enhanced (photo) electrochemical hydrogen evolution. Appl. Surf. Sci..

[24] Josny J., Jinu M., Soney C.G. (2018). Nanomaterials for photoelectrochemical water splitting—Review. Int. J. Hydrog. Energy.

[25] James C.H., Kyoung S.C. (2012). Effect of electrolytes on the selectivity and stability of n-type WO_3_ photoelectrodes for use in solar water oxidation. J. Phys. Chem..

[26] Jason A.S., Kyoung S.C. (2011). Effect of a cobalt-based oxygen evolution catalyst on the stability and the selectivity of photo-oxidation reactions of a WO_3_ photoanode. Chem. Mater..

[27] Qixi M., Almagul Z., Bruce S.B., Harry B.G., Nathan S.L. (2012). A quantitative assessment of the competition between water and anion oxidation at WO_3_ photoanodes in acidic aqueous electrolytes. Energy Environ. Sci..

[28] Emin S., de Respinis M., Fanetti M., Smith W., Valant M., Dam B. (2015). A simple route for preparation of textured WO_3_ thin films from colloidal W nanoparticles and their photoelectrochemical water splitting properties. Appl. Catal. B.

[29] Su J., Feng X., Sloppy J.D., Guo L., Grimes C.A. (2011). Vertically aligned WO₃ nanowire arrays grown directly on transparent conducting oxide coated glass: Synthesis and photoelectrochemical properties. Nano Lett..

[30] Kalanur S.S., Hwang Y.J., Chae S.Y., Joo O.S. (2013). Facile growth of aligned WO_3_ nanorods on FTO substrate for enhanced photoanodic water oxidation activity. J. Mater. Chem. A.

[31] Feng X., Chen Y., Qin Z., Wang M., Guo L. (2016). Facile fabrication of sandwich structured WO_3_ nanoplate arrays for efficient photoelectrochemical water splitting. ACS Appl. Mater. Interfaces.

[32] Wang N., Wang D., Li M., Shi J., Li C. (2014). Photoelectrochemical water oxidation on photoanodes fabricated with hexagonal nanoflower and nanoblock WO_3_. Nanoscale.

[33] Shin S., Han H.S., Kim J.S., Park I.J., Lee M.H., Hong K.S., Cho I.S. (2015). A tree-like nanoporous WO_3_ photoanode with enhanced charge transport efficiency for photoelectrochemical water oxidation. J. Mater. Chem. A.

[34] Fàbrega C., López S., Satoca D., Prades J.D., Alonso M.D., Penelas G., Morante J.R., Andreu T. (2016). Efficient WO_3_ photoanodes fabricated by pulsed laser deposition for photoelectrochemical water splitting with high faradaic efficiency. Appl. Catal. B Environ..

[35] Norton D.P., Eason R. (2007). Pulsed laser deposition of complex materials: Progress toward applications. Pulsed Laser Deposition of Thin Films.

[36] Filipescu M., Ion V., Colceag D., Ossi P.M., Dinescu M. (2012). Growth and characterizations of nanostructured tungsten oxides. Rom. Rep. Phys..

[37] Wang G., Ling Y., Wang H., Yang X., Wang C., Zhang J.Z., Li Y. (2012). Hydrogen-treated WO_3_ nanoflakes show enhanced photostability. Energy Environ. Sci..

[38] Zhao Y., Balasubramanyam S., Sinha R., Lavrijsen R., Verheijen M., Bol A.A., Hutter B.A. (2018). Physical and chemical defects in WO_3_ thin films and their impact on photoelectrochemical water splitting. ACS Appl. Energy Mater..

[39] Kay A., Cesar I., Gratzel M. (2006). New benchmark for water photooxidation bynanostructured a-Fe_2_O_3_ films. J. Am. Chem. Soc..

[40] Qiu Y., Leung S.F., Zhang Q., Hua B., Lin Q., Wei Z., Tsui K.H., Zhang Y., Yang S., Fan Z. (2014). Efficient photoelectrochemical water splitting with ultrathin films of hematite on three-dimensional nanophotonic structures. Nano Lett..

[41] Pala R.A., Leenheer A.J., Lichterman M., Atwater H.A., Lewis N.S. (2014). Measurementof minority-carrier diffusion lengths using wedge-shaped semiconductor photoelectrodes. Energy Environ. Sci..

[42] Heumann T., Stolica N. (1971). The electrochemical behaviour of tungsten—II. The dissolution of tungsten in NaOH solutions. Electrochim. Acta.

[43] Rania B.J., Kumarb M.P., Ravichandranb S., Ravia G., Ganeshc V., Gudurud R.K., Yuvakkumara R., Honge S.I. (2019). WO3 nanocubes for photoelectrochemical water-splitting applications. J. Phys. Chem. Solids.

[44] Ishihara H., Kannarpady K.G., Khedir R.K., Woo J., Trigwell S., Biris S.A. (2011). A novel tungsten trioxide (WO_3_)/ITO porous nanocomposite for enhanced photo-catalytic water splitting. Phys. Chem. Chem. Phys..

[45] Mai M., Ma X., Zhou H., Ye M., Li T., Ke S., Lin P., Zeng X. (2017). Effect of oxygen pressure on pulsed laser deposited WO_3_ thin films for photoelectrochemical water splitting. J. Alloys Compd..

[46] Zou Y.S., Zhang Y.C., Lou D., Wang H.P., Gu L., Dong Y.H., Dou K., Song X.F., Zeng H.B. (2014). Structural and optical properties of WO_3_ films deposited by pulsed laser deposition. J. Alloys Compd..

[47] Palla P.A., Filipescu M., Schneider W.C., Antohe S., Ossi M.P., Radnóczi G., Dinescu M., Wokaun A., Lippert T. (2016). Direct laser deposition of nanostructured tungsten oxide for sensing applications. J. Phys. D Appl. Phys..

[48] Fujiwara H., Collins R.W. (2018). Fundamental principles and solar cell characterization. Spectroscopic Ellipsometry for Photovoltaics.

[49] Zheng G., Wang J., Liu H., Murugadoss V., Zu G., Che H., Lai C., Li H., Ding T., Gao Q. (2019). Tungsten oxide nanostructures and nanocomposites for photoelectrochemical water splitting. Nanoscale.

[50] Crawford S., Thimsen E., Biswas P. (2009). Impact of different electrolytes on photocatalytic water splitting. J. Electrochem. Soc..

[51] Fekete M., Riedel W., Patti A.F., Spiccia L. (2014). Photoelectrochemical water oxidation by screen printed ZnO nanoparticle films: Effect of pH on catalytic activity and stability. Nanoscale.

[52] Tayyebi A., Soltani T., Lee B.K. (2018). Effect of pH on photocatalytic and photoelectrochemical (PEC) properties of monoclinic bismuth vanadate. J. Colloid Interface Sci..

[53] Yourey J.E., Kurt J.B., Bartlett B.M. (2012). Water oxidation on a CuWO_4_−WO_3_ composite electrode in the presence of [Fe(CN)6]3−: Toward solar Z-scheme water splitting at zero bias. J. Phys. Chem. C.

[54] Raja K.S., Mahajan V.K., Misra M. (2006). Determination of photo conversion efficiency of nanotubular titanium oxide photo-electrochemical cell for solar hydrogen generation. J. Power Sources.

