# Supplementary material for: The Influence of the Structural and Morphological Properties of WO3 Thin Films Obtained by PLD on the Photoelectrochemical Water-Splitting Reaction Efficiency
Source: Nanomaterials (Basel). 2021 Jan 6;11(1):110. doi: 10.3390/nano11010110 (PMC7825140; doi:10.3390/nano11010110)
Supplement: Supplementary file 1 [file nanomaterials-11-00110-s001.pdf]

nanomaterials

MDPI

Supplementary Materials

The Influence of the Structural and Morphological Properties of WO_3 Thin Films Obtained by PLD on the Photoelectrochemical Water-Splitting Reaction Efficiency

Florin Andrei [1,2], Andreea Andrei [1], Ruxandra Birjega [1], Eduard Nicolae Sirjita [1,3], Alina Irina Radu [1,3], Maria Dinescu [1], Valentin Ion [1], Valentin-Adrian Maraloiu [4], Valentin Şerban Teodorescu [4] and Nicu Doinel Scarisoreanu [1,*]

1. National Institute for Laser, Plasma and Radiation Physics, 077125 Magurele, Romania; florin.andrei@inflpr.ro (F.A.); andreea.chis@inflpr.ro (A.A.); ruxandra.birjega@inflpr.ro (R.B.); eduard.sirjita@inflpr.ro (E.N.S.); alina.calugar@inflpr.ro (A.I.R.); maria.dinescu@inflpr.ro (M.D.); valentin.ion@inflpr.ro (V.I.)
2. Faculty of Chemistry, University of Bucharest, 030018 Bucharest, Romania
3. Faculty of Physics, University of Bucharest, 077125 Magurele, Romania
4. National Institute for Material Physics, 077125 Magurele, Romania; maraloiu@infim.ro (V. A. M.); teoval@infim.ro (V.S.T.)

* Correspondence: nicu.scarisoreanu@inflpr.ro; Tel.: +40-743147427

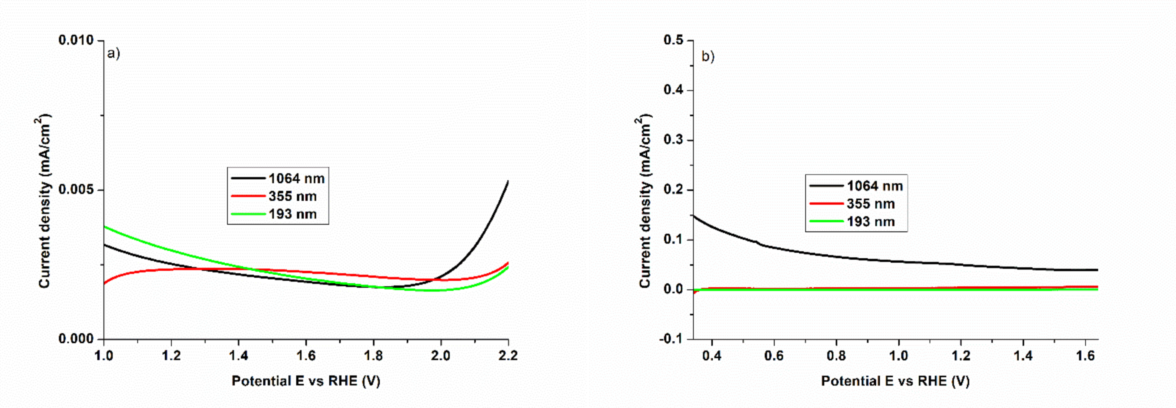

(**a**) (**b**)

Figure S1. Linear sweep voltammetry without irradiation on WO_3/PtSi thin films manufactured at different laser wavelengths in **a**) alkaline electrolyte: NaOH and **b**) acidic electrolyte: H_2SO_4.

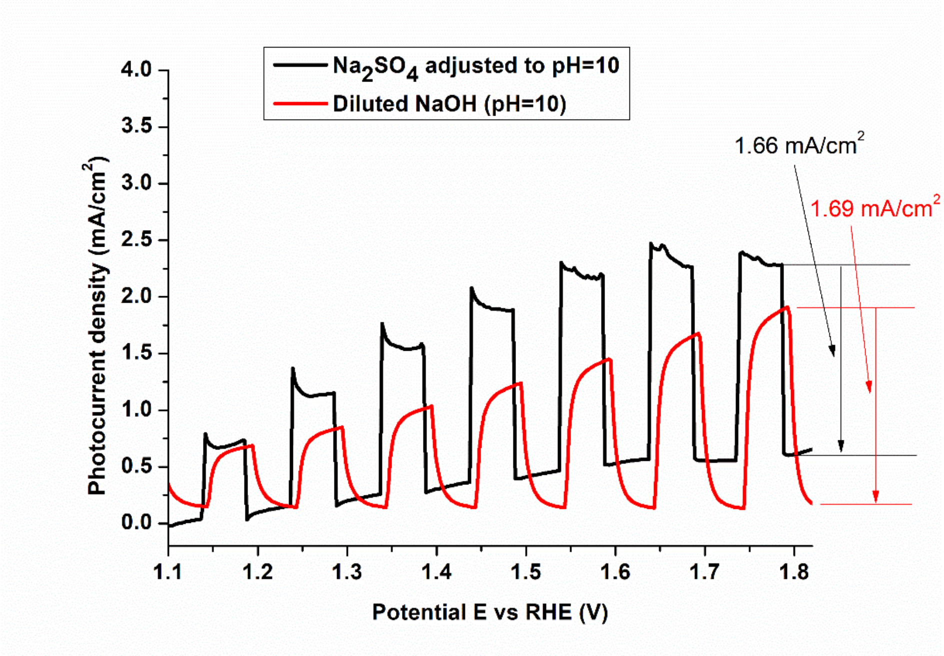

Figure S2. Potentiondynamic measurements under chopped irradiation on WO_3/PtSi thin films manufactured at 1064 nm laser wavelengths in different concentration of alkaline electrolytes.

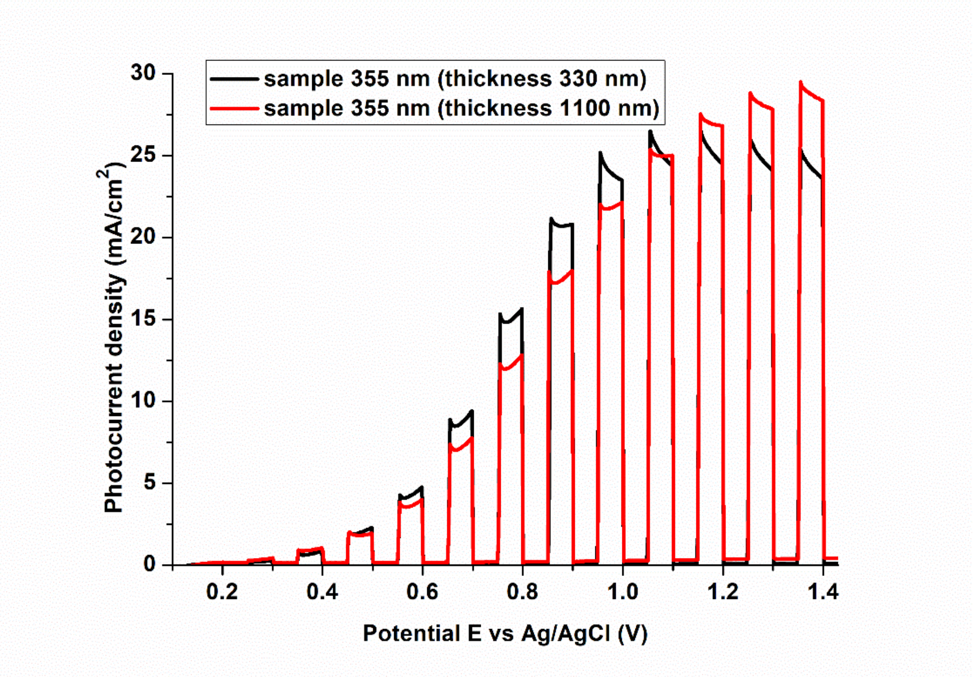

Figure S3. Potentiondynamic measurements under chopped irradiation on WO_3/PtSi thin films manufactured at 355 nm laser wavelengths with different thickeness.

Table S1. *APCE* at 404 nm for WO_3/PtSi thin films.

Sample	APCE 1.2 V vs RHE	APCE 1.4 V vs RHE	APCE 1.6 V vs RHE
1064 nm (alkaline)	0.0031	0.0047	0.0065
355 nm (alkaline)	0.0037	0.0062	0.0094
193 nm (alkaline)	0.005	0.006	0.008
1064 nm (acidic)	0.0331	0.0326	0.0334
355 nm (acidic)	0.077	0.097	0.104
193 nm (acidic)	0.0053	0.0073	0.009

The efficiencies were calculated by using the following formula:

$$APCE = \frac{J(mA \cdot cm^{-2}) \times 1239.8\ (V \cdot nm)}{P_{\lambda}(mW \cdot cm^{-2})\ \times\ \lambda(nm)\ \times A}$$

with the absorptance calculated as shown below from the extinction coefficients (k) measured by spectroscopic ellipsometry and the film thickness (t) calculated by XTEM: $A = 1 - e^{-\frac{4\pi k \cdot t}{\lambda}}$. The incident power density was calculated by taking into account the incident laser diode at the film surface (4.4 mW) and the spot area (0.5 mm^2).

© 2021 by the authors. Licensee MDPI, Basel, Switzerland. This article is an open access article distributed under the terms and conditions of the Creative Commons Attribution (CC BY) license (http://creativecommons.org/licenses/by/4.0/).
